# Short-term effects of ambient PM₂.₅ exposure on cardiovascular mortality in Almaty, Kazakhstan

**DOI:** 10.3389/fpubh.2026.1950463

**Published:** 2026-10-23

**Authors:** Gulnara Kulkayeva, Gulnara Kapanova, Maksat Mamyrkul, Lyazzat Kosherbayeva, Riza Yessergeneva

**Affiliations:** 1 Salidat Kairbekova National Scientific Research Center for Health Development, Ministry of Healthcare of the Republic of Kazakhstan, Astana, Kazakhstan; 2 Farabi Kazakh National University, Almaty, Kazakhstan

**Keywords:** air pollution, cardiovascular mortality, distributed lag nonlinear model, generalized additive model, Kazakhstan

## Abstract

**Background:**

Ambient fine particulate matter (PM₂.₅) is an established environmental risk factor for cardiovascular disease, yet evidence regarding its short-term effects on cardiovascular mortality remains limited in Central Asia. This study investigated the short-term association between PM₂.₅ exposure and cardiovascular mortality in Almaty, Kazakhstan.

**Methods:**

We conducted a retrospective ecological time-series study using daily cardiovascular mortality and environmental data collected between January 1, 2017 and December 31, 2025 (3,287 days). Distributed lag nonlinear models within a generalized additive model framework with a quasi-Poisson distribution were used to estimate lag-specific (0–7 days) and cumulative associations between daily PM₂.₅ concentrations and cardiovascular mortality while adjusting for long-term trends, day of the week, and nonlinear temperature effects. Stratified analyses were performed by age and sex.

**Results:**

During the study period, 43,095 cardiovascular deaths were recorded. The mean daily PM₂.₅ concentration was 36.9 ± 33.3 μg/m^3^. Neither lag-specific nor cumulative associations between PM₂.₅ exposure and cardiovascular mortality reached statistical significance. Relative risk estimates increased after exposure, peaking approximately 2–4 days later before declining. The cumulative relative risk for the overall population was 1.011 (95% CI, 0.837–1.222). Adults aged ≥65 years exhibited the highest cumulative relative risk estimate (RR 1.174, 95% CI, 0.926–1.489), although all subgroup confidence intervals included the null value.

**Conclusion:**

Short-term PM₂.₅ exposure was not significantly associated with cardiovascular mortality in Almaty; however, the observed delayed temporal pattern was biologically plausible and suggested greater susceptibility among older adults.

## Introduction

Cardiovascular diseases (CVD) remain the leading cause of death globally, accounting for an estimated 19.8 million deaths in 2022, or approximately 32% of all global deaths (1). Despite advances in prevention and treatment, CVDs continue to pose a major public health challenge, particularly in low- and middle-income countries. In addition to established cardiovascular risk factors, ambient air pollution has emerged as an important environmental determinant of cardiovascular morbidity and mortality (2).

Ambient air pollution, particularly fine particulate matter (PM₂.₅), has emerged as an important modifiable environmental risk factor for CVD (3). A growing body of epidemiological evidence indicates that both short- and long-term exposure to PM₂.₅ are associated with increased risks of myocardial infarction, stroke, heart failure, arrhythmias, and cardiovascular mortality, with adverse effects observed even at concentrations below current air quality guidelines (4). These associations are biologically plausible and are thought to be mediated through oxidative stress, systemic inflammation, endothelial dysfunction, autonomic imbalance, and increased thrombogenicity (5). Older adults and individuals with pre-existing cardiovascular disease appear to be particularly susceptible to these adverse effects, whereas healthy lifestyle behaviors may partially reduce pollution-related cardiovascular risk (3, 4).

Despite increasing evidence from North America, Europe, and East Asia, epidemiological data on the short-term cardiovascular effects of PM₂.₅ exposure remain scarce in Central Asia (6). Kazakhstan experiences substantial air pollution, with previous studies estimating a significant burden of premature mortality attributable to PM₂.₅ exposure and emphasizing the need for region-specific evidence to support environmental and public health policies (6). Almaty, the largest city in Kazakhstan, experiences severe seasonal PM₂.₅ pollution, particularly during the cold season, because of domestic heating, traffic emissions, unfavorable topography, and frequent temperature inversions (7). However, the short-term association between ambient PM₂.₅ exposure and cardiovascular mortality has not been systematically investigated in this population.

The aim of this study was to investigate the short-term association between ambient PM₂.₅ exposure and cardiovascular mortality in Almaty, Kazakhstan, using distributed lag nonlinear models within a generalized additive model framework. We also evaluated lag-specific and cumulative effects of PM₂.₅ exposure and explored potential differences according to age and sex.

## Methods

*Study design*. A retrospective ecological time-series study was conducted to investigate the short-term association between ambient PM₂.₅ concentrations and daily cardiovascular mortality (CVM) in Almaty, Kazakhstan. The study covered the period from January 1, 2017 to December 31, 2025, comprising 3,287 consecutive daily observations.

*Mortality data*: Daily records of CVM were obtained from the National Research Center for Health Development of the Republic of Kazakhstan (Almaty branch). Causes of death were classified according to the International Classification of Diseases, Tenth Revision (ICD-10), Chapter IX (Diseases of the Circulatory System). During the study period, a total of 43,095 cardiovascular deaths were recorded. Individual records included the date of death, sex, age, and the underlying cause of death. Daily counts of cardiovascular deaths were calculated for the overall population and were further stratified by sex (male and female) and age (<65 years and ≥65 years) to evaluate potential effect modification. Only anonymized aggregated daily mortality counts were analyzed; therefore, no personally identifiable information was available.

*Air pollution and meteorological data:* PM₂.₅ exposure was assessed at the city-day level. Available PM₂.₅ monitoring data for Almaty were used, with the number of monitoring locations contributing measurements varying over the study period according to data availability. Detailed information on the exact number and locations of individual monitoring stations was not available in the dataset. For each calendar day, all available valid PM₂.₅ measurements (μg/m^3^) were pooled and averaged to derive a single daily mean PM₂.₅ concentration.

Daily mean ambient temperature (°C) was collected from the corresponding meteorological monitoring stations and included in the analysis as a potential confounding variable.

*Statistical analysis:* The association between short-term PM₂.₅ exposure and daily CVM was evaluated using Distributed Lag Nonlinear Models (DLNMs) within a Generalized Additive Model (GAM) framework with a quasi-Poisson distribution to account for overdispersion in daily mortality counts.

The expected number of daily cardiovascular deaths was modeled as:

$$\begin{array}{l} \log[E(Y_{t})]=\alpha +\mathrm{cb}\;(\mathrm{PM}₂._{5}{,}_{t})+\mathrm{cb}\;({\text{Temperature}}_{t})\\ +s\;(\mathrm{Tim}e_{t})+\mathrm{DOW}\;t \end{array}$$

where Y*_t_*—expected number of CVM on day t; cb (PM₂.₅)—cross-basis function describing both the exposure–response relationship and distributed lag structure for PM₂.₅_;_ cb (Temperature)—cross-basis function for daily mean temperature; s (Time)—penalized cubic regression spline controlling for long-term and seasonal temporal trends; DOW represents categorical indicator variables for day of the week.

Long-term and seasonal trends were controlled using a penalized cubic regression spline with seven degrees of freedom per year. Daily mean temperature was modeled using a natural cubic spline with four degrees of freedom and a lag period of 3 days to account for delayed nonlinear meteorological effects.

*Lag-response modeling:* To evaluate delayed health effects, PM₂.₅ exposure was modeled across lag days 0–7, reflecting acute cardiovascular responses reported in previous environmental epidemiology studies.

The PM₂.₅ cross-basis matrix was specified using a linear exposure function combined with a natural cubic spline (3 degrees of freedom) for the lag dimension. Relative risks (RR) with corresponding 95% confidence intervals (95% CIs) were estimated for each lag day and for the cumulative exposure over the entire 7-day lag period.

*Sensitivity analyses*: To assess the robustness of the findings, sensitivity analyses were conducted using alternative model specifications. The maximum lag period for PM₂.₅ was varied from the primary lag of 0–7 days to 0–5 and 0–10 days. The degrees of freedom used to control for long-term and seasonal temporal trends were varied from 7 df per year in the primary model to 6 and 8 df per year. The degrees of freedom for daily mean temperature were varied from 4 df in the primary model to 3 and 5 df. Effect estimates obtained from these alternative specifications were compared with those from the primary model. Additional sensitivity analyses: Additional sensitivity analyses were performed to evaluate the potential influence of other environmental factors. These analyses included additional adjustments for relative humidity, precipitation, wind speed, atmospheric pressure, and NO₂. Because these environmental data were available only from 2 December 2021 to 31 December 2025, the additional analyses were restricted to this period. Models including NO₂ were based on 1,384 daily observations because of incomplete NO₂ measurements. O₃ was not included because data were not available throughout the study period. To further examine seasonal variation in the association between PM₂.₅ exposure and cardiovascular mortality, additional analyses were conducted separately for winter, spring, summer, and autumn. Season-specific relative risks and corresponding 95% confidence intervals were estimated.

*Stratified analyses:* Potential effect modification was evaluated by repeating all DLNM analyses separately for males, females, individuals younger than 65 years, and those aged 65 years or older. Lag-specific and cumulative relative risks were estimated for each subgroup.

All statistical analyses were performed using R software. Generalized additive models were fitted using the mgcv package, whereas distributed lag nonlinear models were implemented using the dlnm package. Statistical significance was defined as a two-sided *p-*value < 0.05.

## Results

*Descriptive characteristics of the study population:* During the study period (January 2017 to December 2025), a total of 43,095 cardiovascular deaths were recorded over 3,287 observation days. Males accounted for 24,082 cases (55.9%), whereas females accounted for 19,013 cases (44.1%). Individuals aged ≥65 years represented 60.6% of all CVM, while 39.4% occurred among individuals younger than 65 years (Table 1). The median daily PM₂.₅ concentration during the study period was 21.7 μg/m^3^ (IQR: 12.4–47.2 μg/m^3^), with daily concentrations ranging from 3.1 to 263.7 μg/m^3^. The median daily ambient temperature was 11.8 °C (IQR: 1.3–20.7 °C), ranging from −25.5 °C to 31.3 °C (Table 1).

**Table 1:** Baseline descriptive statistics of cardiovascular events and daily environmental variables in Almaty (2017–2025).

Parameter/Variable		Number of cases (N)	Percentage (%)	Median (IQR)	Minimum–maximum
Cardiovascular events (total)		43,095	100.0	—	—
Gender	Male	24,082	55.9	—	—
	Female	19,013	44.1	—	—
Age groups	Young (< 65 years old)	16,979	39.4	—	—
	Older adults	26,116	60.6	—	—
Environmental exposures (daily)		3,287 days	—	—	—
PM₂.₅ (μg/m^3^)		—	—	21.7 (12.4–47.2)	3.1–263.7
Temperature (°C)		—	—	11.8 (1.3–20.7)	−25.5 – 31.3

*N*, number of cases; SD, standard deviation; IQR, interquartile range; PM₂.₅, particulate matter with an aerodynamic diameter ≤2.5 μm.

*Seasonal variation in PM₂.₅ concentrations:* Marked seasonal variability in PM₂.₅ concentrations was observed throughout the study period. Monthly mean concentrations exceeded 75 μg/m^3^ during January and February and remained elevated in December, whereas the lowest concentrations were observed during late spring and summer (May–August), when monthly averages ranged from 13.0 to 14.6 μg/m^3^ (Table 2).

**Table 2:** Monthly variations in PM_2.5_ concentrations and ambient temperature in Almaty (2017–2025).

Month	PM₂.₅ Mean (μg/m^3^)	PM₂.₅ Standard deviation (SD)	Temperature mean (°C)	Temperature standard deviation (SD)
January	87.6	42.6	−3.8	3.0
February	75.5	31.8	−1.0	1.8
March	35.8	13.9	6.4	1.7
April	22.4	8.7	13.3	1.7
May	14.5	5.3	18.3	1.3
June	13.0	3.3	23.4	1.2
July	14.6	6.0	26.4	1.1
August	14.1	5.8	24.2	1.0
September	17.2	8.5	18.4	1.6
October	32.0	16.1	10.9	1.4
November	50.2	26.0	3.1	2.4
December	68.4	35.2	−2.1	2.4

SD, standard deviation; PM₂.₅, particulate matter with an aerodynamic diameter ≤2.5 μm.

The seasonal boxplot (Figure 1) confirmed substantially higher PM₂.₅ concentrations during winter compared with the remaining seasons, accompanied by considerably greater variability and more frequent extreme pollution episodes. Summer demonstrated the lowest concentrations and the narrowest distribution of daily PM₂.₅ values.

**Figure 1:**
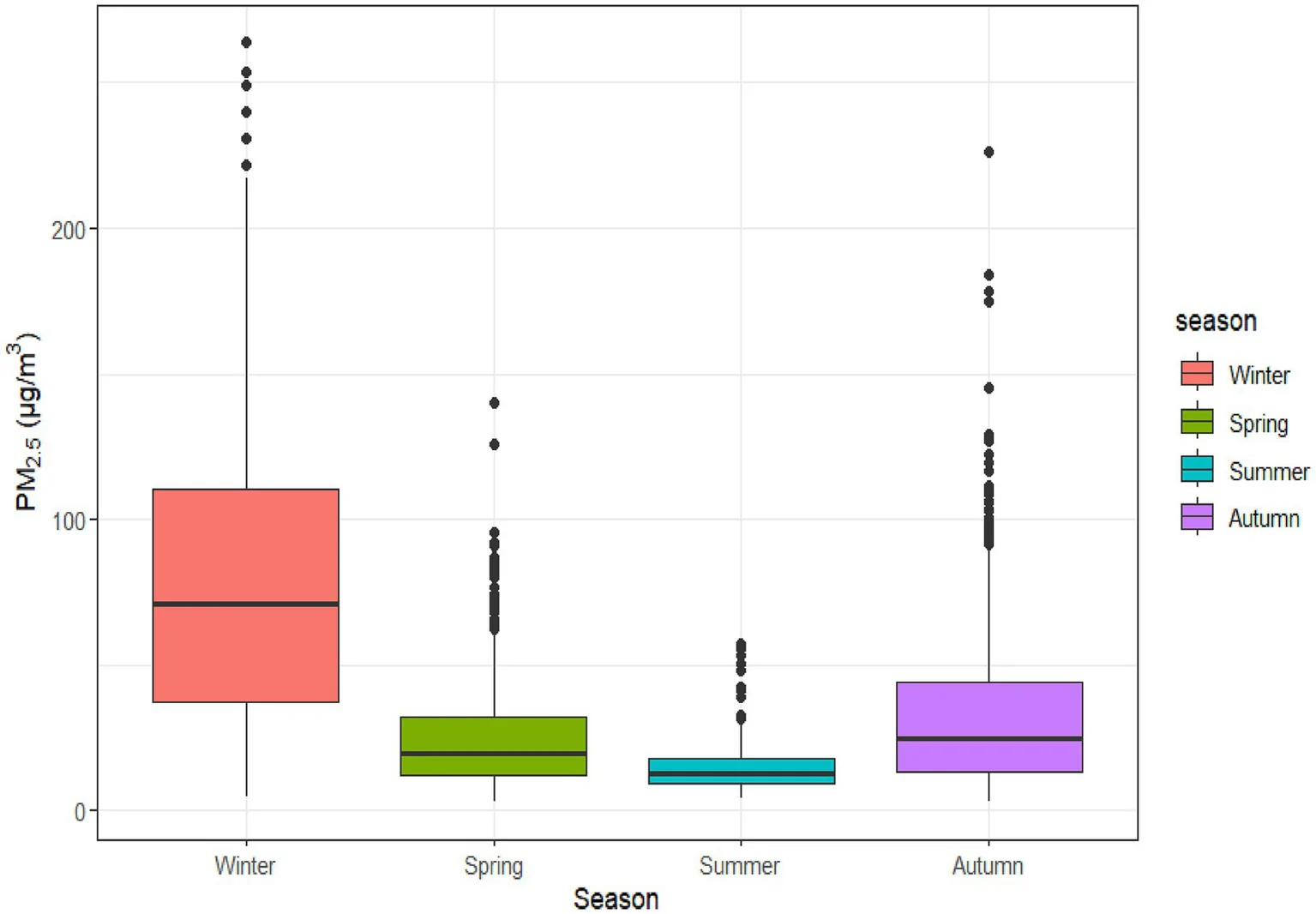
Seasonal distribution of daily PM_2.5_ concentrations in Almaty (2017–2025).

*Distributed lag nonlinear association between* PM₂.₅ *exposure and CVM:* The distributed lag nonlinear model demonstrated that the association between PM₂.₅ exposure and CVM varied according to both exposure concentration and lag duration.

Lag-specific estimates showed that RR increased gradually following PM₂.₅ exposure, with the highest point estimates were observed approximately 3–4 days after exposure. For the overall study population, the estimated RR increased from 1.016 (95% CI: 0.948–1.089) at Lag 1 to 1.043 (95% CI: 0.952–1.142) at Lag 3 before gradually declining during subsequent lag days (Table 3).

**Table 3:** Lag-specific relative risks and 95% confidence intervals for cardiovascular events at a PM_2.5_ concentration of 100 μg/m^3^.

Lag period	All cases	Male	Female	Older adults (≥65)	Young (<65)
Lag 0 (smog day)	0.991 (0.851–1.155)	0.989 (0.806–1.213)	0.999 (0.797–1.253)	1.106 (0.909–1.345)	0.838 (0.660–1.065)
Lag 1 (1st day)	1.016 (0.948–1.089)	1.026 (0.935–1.125)	1.009 (0.911–1.118)	1.074 (0.983–1.173)	0.936 (0.839–1.043)
Lag 2 (2nd day)	1.035 (0.979–1.095)	1.055 (0.978–1.137)	1.017 (0.936–1.105)	1.044 (0.972–1.122)	1.026 (0.938–1.121)
Lag 3 (3rd day)	1.043 (0.952–1.142)	1.064 (0.942–1.203)	1.022 (0.892–1.170)	1.019 (0.907–1.146)	1.085 (0.939–1.255)
Lag 4 (4th day)	1.033 (0.940–1.134)	1.045 (0.921–1.186)	1.021 (0.887–1.174)	1.000 (0.886–1.128)	1.090 (0.939–1.264)
Lag 5 (5th day)	1.005 (0.947–1.068)	0.998 (0.921–1.082)	1.014 (0.927–1.109)	0.986 (0.913–1.065)	1.037 (0.944–1.141)
Lag 6 (6th day)	0.968 (0.918–1.020)	0.936 (0.873–1.004)	1.004 (0.928–1.085)	0.975 (0.912–1.043)	0.953 (0.878–1.035)
Lag 7 (7th day)	0.926 (0.817–1.049)	0.870 (0.736–1.027)	0.992 (0.823–1.194)	0.966 (0.823–1.134)	0.861 (0.707–1.048)

Values are presented as RR (95% CI). RR, relative risk; CI, confidence interval; PM₂.₅, particulate matter with an aerodynamic diameter ≤2.5 μm.

The exposure–lag contour plot (Figure 2) demonstrated progressively increasing RR at higher PM₂.₅ concentrations and longer lag periods, with the highest predicted risks occurring approximately 3–6 days after exposure. However, confidence intervals widened at the highest concentrations, indicating greater uncertainty in regions with fewer observations.

**Figure 2:**
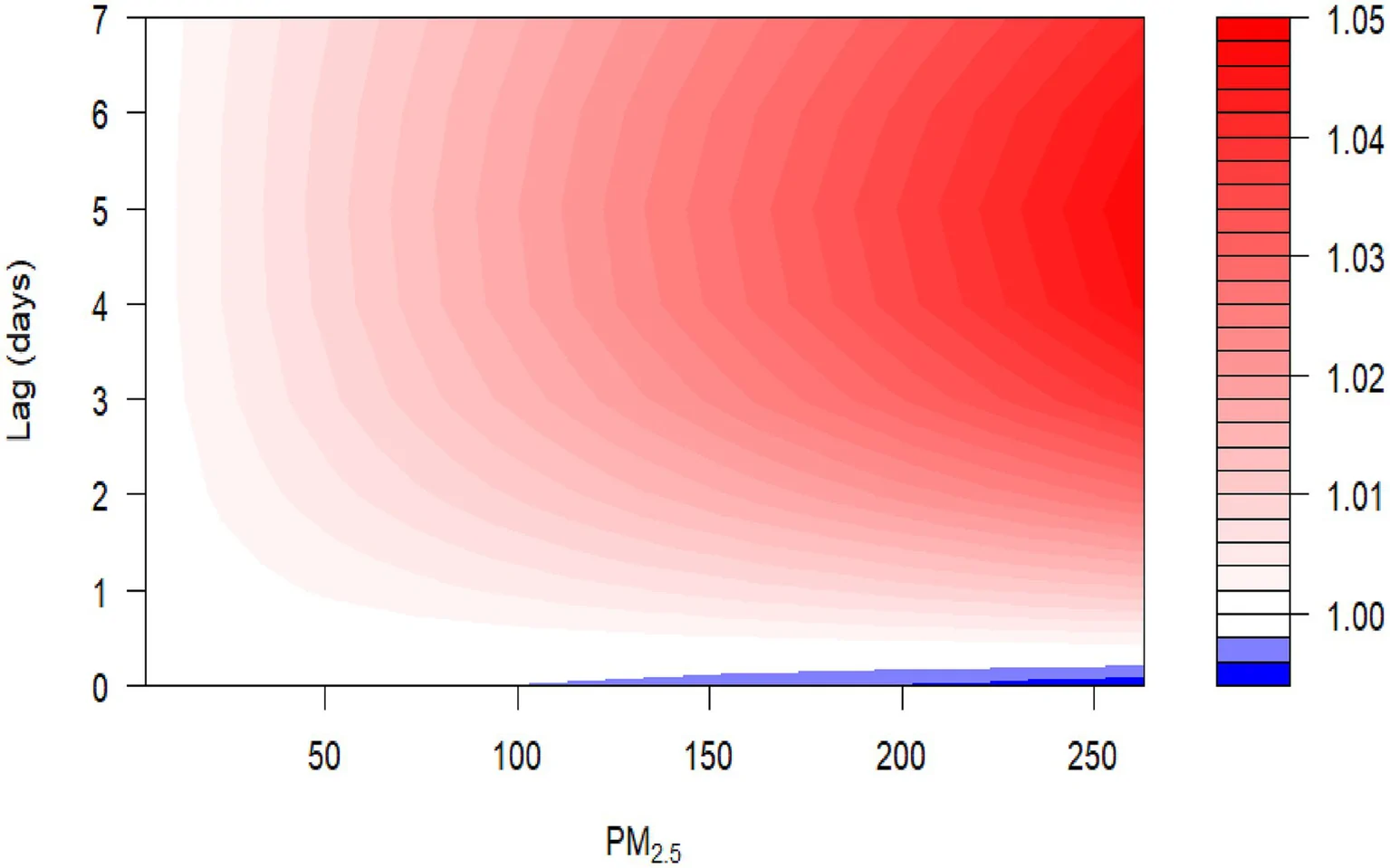
Contour plot illustrating the exposure–lag–response relationship between PM_2.5_ concentration and cardiovascular mortality estimated using the DLNM.

The lag-response curve (Figure 3) showed a similar temporal pattern, with RR remaining close to unity immediately after exposure and peaking during intermediate lag days before returning toward baseline by the end of the 7-day lag period.

**Figure 3:**
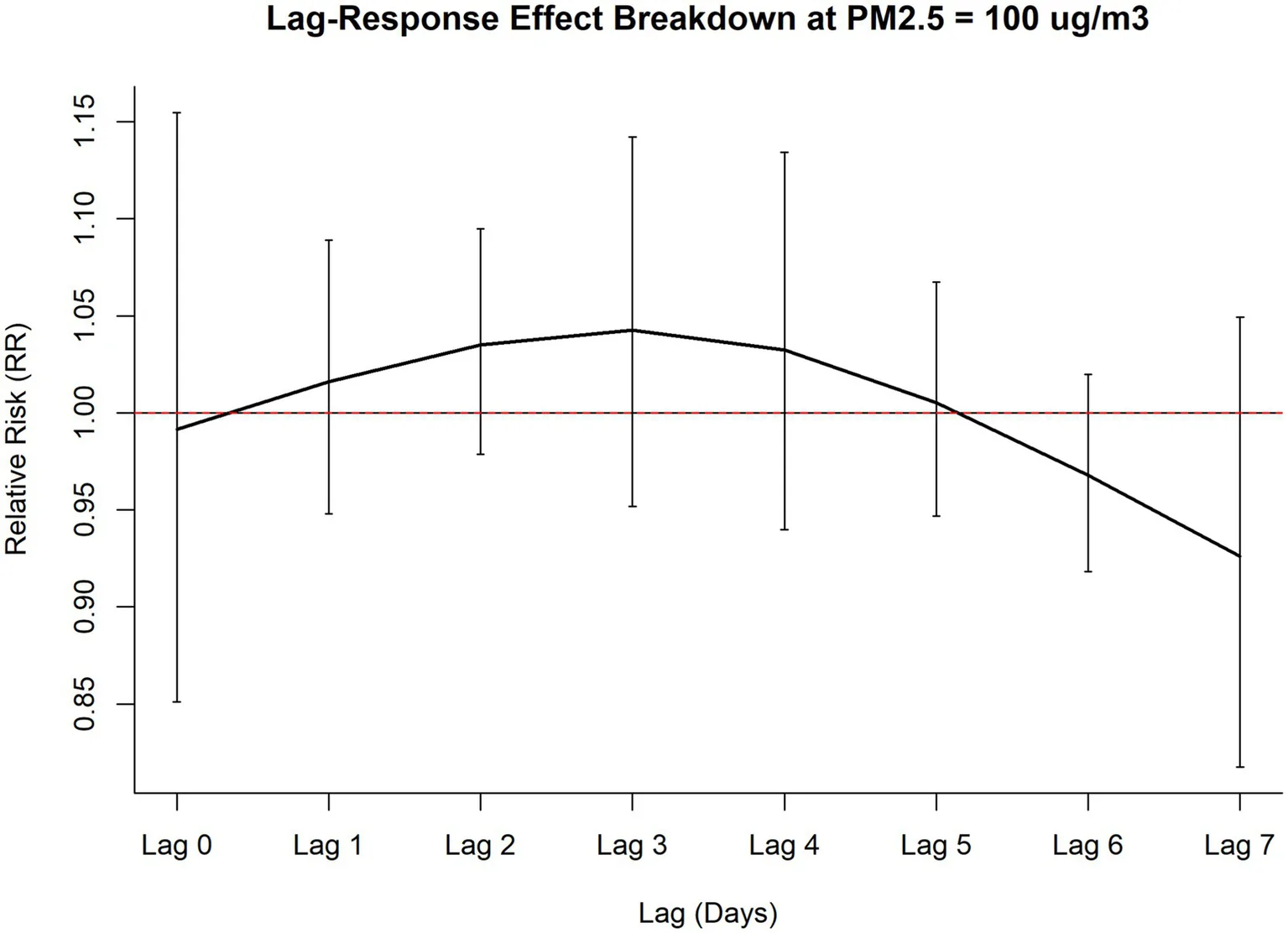
Lag-specific relative risks (95% CI) associated with PM_2.5_ exposure at 100 μg/m^3^.

*Cumulative effects of* PM₂.₅ *exposure:* The cumulative effect estimated over the entire 0–7 day lag period suggested a modest positive association between PM₂.₅ exposure and CVM. For the overall population, the cumulative RR was 1.011 (95% CI: 0.837–1.222), suggesting a small increase in CVM that did not reach statistical significance (Table 4).

**Table 4:** Total cumulative relative risks (cumulative RR) over a 7-day lag window.

Patient subgroup	Cumulative RR	95% CI
All Cases	1.011	(0.837–1.222)
Male	0.966	(0.756–1.235)
Female	1.079	(0.819–1.423)
Older adults (≥65 years)	1.174	(0.926–1.489)
Young (< 65 years)	0.810	(0.606–1.082)

RR, relative risk; CI, confidence interval.

*Stratified analyses:* Subgroup analyses demonstrated differences in susceptibility across demographic groups (Table 4). The highest cumulative RR was observed among individuals aged 65 years and older (RR = 1.174, 95% CI: 0.926–1.489), whereas younger individuals exhibited lower cumulative estimates (RR = 0.810, 95% CI: 0.606–1.082). Female participants showed slightly higher cumulative risk estimates (RR = 1.079, 95% CI: 0.819–1.423) than males (RR = 0.966, 95% CI: 0.756–1.235). However, confidence intervals overlapped unity in all subgroup analyses.

Overall, subgroup analyses suggested that older adults (≥65 years) may be more susceptible to the delayed effects of PM₂.₅ exposure on cardiovascular mortality than younger individuals. However, all confidence intervals included unity, and therefore these findings should be interpreted cautiously as they did not reach statistical significance.

*Sensitivity analyses:* The estimated association between PM₂.₅ exposure and cardiovascular mortality remained broadly consistent across alternative model specifications. Changing the PM₂.₅ lag window from 0–7 days to 0–5 and 0–10 days and varying the degrees of freedom for temporal trends from 7 to 6 and 8 df/year did not materially alter the direction or magnitude of the effect estimates. Similarly, varying the degrees of freedom for temperature from 4 to 3 and 5 produced broadly consistent estimates. Additional sensitivity analyses: Additional adjustment for environmental covariates did not materially alter the estimated association between PM₂.₅ and cardiovascular mortality. The RR was 1.021 (95% CI: 0.957–1.090) in the main model for the restricted analysis period and remained similar after adjustment for relative humidity (RR 1.020, 95% CI: 0.956–1.089), precipitation (RR 1.020, 95% CI: 0.956–1.089), wind speed (RR 1.022, 95% CI: 0.958–1.091), atmospheric pressure (RR 1.009, 95% CI: 0.945–1.079), and NO₂ (RR 1.001, 95% CI: 0.936–1.071). The fully adjusted model including all available meteorological variables and NO₂ yielded an RR of 0.989 (95% CI: 0.923–1.060). Seasonal analysis: Season-specific analyses showed an RR of 0.988 (95% CI: 0.542–1.800) in winter, 1.388 (95% CI: 0.875–2.204) in spring, 0.729 (95% CI: 0.227–2.339) in summer, and 0.984 (95% CI: 0.648–1.495) in autumn. Although the point estimates varied across seasons, all confidence intervals included the null value and were relatively wide; therefore, the seasonal differences should be interpreted cautiously (Supplementary material 1).

## Discussion

We found no statistically significant lag-specific or cumulative association between PM₂.₅ exposure and cardiovascular mortality. However, the analyses identified a consistent temporal pattern characterized by increasing relative risks during lag days 2–4, followed by a gradual decline. Older adults (≥65 years) exhibited the highest cumulative relative risk estimate, although the precision of the subgroup estimates was limited.

The delayed increase in relative risk estimates during lag days 2–4 is biologically plausible. Following inhalation, PM₂.₅ can initiate a cascade of biological responses, including oxidative stress and inflammation, which may subsequently lead to endothelial dysfunction, autonomic nervous system imbalance, increased blood coagulability, and destabilization of atherosclerotic plaques (8). Together, these mechanisms may increase the risk of fatal cardiovascular events several days after exposure rather than immediately. Consistent with this concept, previous studies have shown that PM₂.₅ related changes in inflammatory biomarkers, such as high-sensitivity C-reactive protein, occur after several days of exposure, suggesting that the timing of biological responses varies according to the underlying pathophysiological process. The subsequent decline in relative risk estimates after approximately 4 days is also compatible with the transient nature of these acute physiological responses (9, 10).

The temporal pattern observed in the present study is generally consistent with previous studies indicating that short-term cardiovascular effects of PM₂.₅ may vary across lag days following exposure (11, 12). Nevertheless, the magnitude of the associations in our study was modest, and all confidence intervals included the null value. Differences between published findings may reflect variation in population characteristics, baseline cardiovascular risk, exposure assessment methods, meteorological conditions, healthcare systems, statistical modeling approaches, and the chemical composition of PM₂.₅. Because PM₂.₅ toxicity depends on its chemical constituents, equal mass concentrations do not necessarily produce equivalent health effects across different geographic settings (13).

The environmental setting of Almaty is an important consideration when interpreting the present findings. The city is situated in a mountain basin where weak atmospheric ventilation and frequent temperature inversions during the cold season promote the accumulation of air pollutants. Consequently, prolonged winter smog episodes are common, with markedly elevated PM₂.₅ concentrations during January, February, and December, largely driven by domestic heating, traffic emissions, and limited atmospheric dispersion (7, 14). In contrast, substantially lower PM₂.₅ concentrations are typically observed during late spring and summer when meteorological conditions favor pollutant dispersion. These distinctive seasonal and meteorological characteristics may influence not only the magnitude and timing of population exposure but also the chemical composition of PM₂.₅, potentially contributing to differences in health effects compared with those reported in other urban settings (13).

Exposure misclassification is another possible explanation for the imprecise findings. Daily concentrations obtained from fixed-site monitoring stations represent area-level ambient exposure and may not accurately reflect individual exposure. Differences in residential location, time spent indoors, occupational exposure, heating practices, building ventilation, and personal mobility could not be considered. Such non-differential exposure error would generally be expected to attenuate associations towards the null (15). Although daily time-series designs control for stable individual characteristics by design, residual confounding from time-varying factors may remain. Future epidemiological studies in Almaty may benefit from expanding the spatial coverage of ambient PM₂.₅ monitoring networks and integrating wearable personal air pollution sensors to improve individual exposure assessment, reduce exposure misclassification, and increase the precision of estimated health effects (16–18).

The higher cumulative RR estimate among adults aged ≥65 years may indicate greater susceptibility in older populations; however, the wide confidence interval indicates substantial uncertainty in this subgroup estimate. Older adults are more likely to have pre-existing cardiovascular disease, impaired vascular function, reduced physiological reserve, and multiple comorbid conditions, which may increase vulnerability to air pollution. The apparent difference between older and younger individuals should therefore be considered suggestive rather than confirmatory. Similarly, the slightly higher cumulative estimate among females than males does not establish sex-specific effect modification because the subgroup confidence intervals overlapped substantially (19, 20).

The findings remain relevant from a public health perspective despite the lack of statistical significance. Almaty experiences recurrent episodes of high PM₂.₅ exposure that affect a large urban population. Even a small increase in cardiovascular mortality during severe pollution episodes could result in a meaningful population burden when exposure is widespread and repeated over time (8). Measures aimed at reducing emissions from heating, combustion sources, and transport, as well as improving public warning systems during severe pollution episodes, may therefore provide health benefits. Older adults and individuals with established cardiovascular disease may represent priority groups for targeted risk communication and preventive interventions.

*Strengths and limitation:* This study has several strengths. It included a long, continuous observation period of 9 years, a large number of cardiovascular deaths, and daily environmental and mortality data. The use of a distributed lag nonlinear model allowed the temporal pattern of the association to be evaluated across several days following exposure. The analysis also accounted for overdispersion, long-term and seasonal trends, day of the week, and delayed nonlinear temperature effects. In addition, stratified analyses provided preliminary information regarding possible differences by age and sex.

Several limitations should also be acknowledged. First, the ecological design does not allow individual-level causal inference. Second, ambient concentrations from monitoring stations may not represent personal exposure. Third, information on indoor pollution, smoking, comorbidities, socioeconomic conditions, and medication use was unavailable. Fourth, residual confounding from other pollutants, infectious disease patterns, humidity, holidays, and changes in healthcare access cannot be excluded. Fifth, the study period included the COVID-19 pandemic, which may have influenced mortality patterns, population mobility, emissions, and healthcare utilization. Finally, subgroup and high-exposure estimates were imprecise, as reflected by wide confidence intervals. The absence of statistically significant associations requires careful interpretation. It should not be considered evidence that PM₂.₅ has no effect on cardiovascular mortality. Rather, the wide confidence intervals indicate uncertainty regarding the direction and magnitude of the estimated associations. Several factors may have reduced statistical precision. Although the study included 43,095 cardiovascular deaths over 3,287 observation days, the single-city design and the relatively limited number of daily cardiovascular deaths may have reduced the statistical power to detect modest short-term associations. This limitation was particularly relevant to the subgroup analyses, which involved smaller numbers of events and consequently greater statistical uncertainty.

## Conclusion

The present study identified a delayed temporal pattern in the association between PM₂.₅ exposure and cardiovascular mortality in Almaty, with the highest relative risk estimates occurring approximately 2–4 days after exposure. Although neither the lag-specific nor cumulative associations reached statistical significance, the observed temporal pattern was biologically plausible, and older adults showed the highest cumulative relative risk estimates. Further studies incorporating multipollutant models, alternative lag structures, season-specific analyses, and improved exposure assessment are needed to better characterize the short-term effects of PM₂.₅ on cardiovascular mortality in Almaty and other Central Asian cities.

## Data Availability

The raw data supporting the conclusions of this article will be made available by the authors, without undue reservation.
