# Supplementary material for: Short-term effects of ambient PM₂.₅ exposure on cardiovascular mortality in Almaty, Kazakhstan
Source: Front Public Health. 2026 Oct 23;14:1950463. doi: 10.3389/fpubh.2026.1950463 (PMC13739815; doi:10.3389/fpubh.2026.1950463)
Supplement: Supplementary file 1 [file Data_Sheet_1.docx]

Supplementary Material 1

Additional Sensitivity and Seasonal Analyses
